# New report of two Nemoleontinae species from the Korean Peninsula (Neuroptera, Myrmeleontiformia, Myrmeleontidae)

**DOI:** 10.3897/BDJ.14.e203903

**Published:** 2026-07-06

**Authors:** Jiseung Kim, Jaegwan Yang, Sora Kim

**Affiliations:** 1 Lab. of Insect Phylogenetics and Evolution, Department of Bioenvironmental Chemistry, Jeonju, Republic of Korea Lab. of Insect Phylogenetics and Evolution, Department of Bioenvironmental Chemistry Jeonju Republic of Korea; 2 Department of Agricultural Convergence Technology, Jeonbuk National University, Jeonju, Republic of Korea Department of Agricultural Convergence Technology, Jeonbuk National University Jeonju Republic of Korea

**Keywords:** Bigeumdo, island fauna, new records, *
Paraglenurus
*, *
Pseudoformicaleo
*, Shinan-gun, South Korea

## Abstract

**Background:**

*Pseudoformicaleo* and *Paraglenurus* are genera belonging to the tribes Nemoleontini and Megistopini of Nemoleontinae, respectively. Prior to this study, *Pseudoformicaleo* had not been recorded in the Korean Peninsula, while three species of *Paraglenurus* (*Paraglenurus albiventris* Matsumoto, Kikuta & Hayashi, 2021, *Paraglenurus japonicus* (McLachlan, 1867), *and Paraglenurus melanostictus* Matsumoto, Kikuta & Hayashi, 2021) have been recorded in the country.

**New information:**

In this paper, *Pseudoformicaleo nubecula* (Gerstaecker, 1885) and *Paraglenurus okinawensis* (Okamoto, 1910) are reported from the Korean Peninsula for the first time. A diagnosis and supplementary description of both species are provided, together with illustrations of the adult and genitalia. An updated key to species of Korean Nemoleontinae is also provided.

## Introduction

Myrmeleontidae, commonly known as antlions, and owlflies, is the largest family in the order Neuroptera. This family comprises approximately 2,100 known species worldwide and consists of four subfamilies: Ascalaphinae Lefèbvre, 1842, Dendroleontinae Banks, 1899, Myrmeleontinae Latreille, 1802, and Nemoleontinae Banks, 1911 (Machado et al. 2019, Oswald 2026). Among them, Nemoleontinae is a cosmopolitan subfamily comprising 66 genera and more than 670 species, including four tribes: Glenurini Banks, 1927, Megistopini Navás, 1912, Nemoleontini Banks, 1911, and Protoplectrini Tillyard, 1916 (Machado et al. 2019, Zheng and Liu 2023, Oswald 2026). The genus *Pseudoformicaleo* belongs to the tribe Nemoleontini and was established by van der Weele (1909), based on the type species *Pseudoformicaleon gracilis* (Klug in Ehrenberg, 1834). It consists of eight species worldwide and is widely distributed throughout the Old World (New 1985, Sekimoto 2014, Wang et al. 2018, Balakrishnan et al. 2024, Oswald 2026). The genus *Paraglenurus* belongs to the tribe Megistopini and was also established by van der Weele (1909), based on the type species *Paraglenurus scopifer* (Gerstaecker, 1887). Although originally classified under Nemoleontini, it was reclassified into Megistopini by Zheng et al. (2025). It comprises 13 species worldwide and is distributed across Asia (China, Indonesia, Japan, North Korea, South Korea, Russia (Far east)) and Africa (Madagascar, Seychelles) (Sekimoto 2014, Wang et al. 2018, Ábrahám 2023, Zheng and Liu 2025, Oswald 2026).

In Korea, six species of Nemoleontinae have been recorded. Regarding its taxonomic history, Kuwayama (1924) first recorded *Deutoleon lineatus lineatus* (Fabricius) based on specimens collected from Pyongyang and other regions. Subsequently, Okamoto (1926) recorded three species: *Distoleon contubernalis* (McLachlan, 1875), *Distoleon nigricans* (Okamoto, 1910), and *Paraglenurus japonicus* (McLachlan, 1867). Most recently, Kim et al. (2025) reidentified *Distoleon contubernalis* (McLachlan, 1875) as *Distoleon littoralis* Miller & Stange, 1999, and newly recorded two species, *Paraglenurus albiventris* Matsumoto, Kikuta & Hayashi, 2021, and *Paraglenurus melanostictus* Matsumoto, Kikuta & Hayashi, 2021, in Korea.

In this paper, *Pseudoformicaleo nubecula* (Gerstaecker, 1885) and *Paraglenurus okinawensis* (Okamoto, 1910) are reported from the Korean Peninsula for the first time. Comprehensive description and illustration of the species are provided. Additionally, to facilitate identification, we provide a taxonomic key to the species of the subfamily Nemoleontinae, including the two newly recorded species.

## Materials and methods

Specimens were obtained from Bigeumdo Island, Shinan-gun by direct collections of adults. Samples were collected using insect nets and light traps. All specimens examined in this study are preserved dry and deposited at Jeonbuk National University (JBNU, Jeonju, South Korea).

Morphological terminology follows Sekimoto (2014), Breitkreuz et al. (2017), Wang et al. (2018), and Machado and Oswald (2020). The abbreviations used for wing veins: 1A, 2A, 3A – anal veins; CuA – cubitus anterior; CuP – cubitus posteroir; MA – media anterior; MP – media posterior; RA – radius anterior; RP – radius posterior. The abbreviations used for adult size measurements: BL – body length from the front of the head to the tip of the abdomen; FWL – forewing length from its base to apex; HWL – hindwing length from its base to apex. Genital preparations were made with 10% KOH at 70°C for 20 min (Hayashi et al. 2020). After rinsing the KOH with distilled water, the apex of the abdomen was transferred to glycerin for further examination (Zheng et al. 2022).

The specimens were observed using a Canon EOS 6D camera (Canon, Japan) with a Canon Macro Lens EF 100mm (Canon, Japan). Dissected genitalia were observed using a Tucsen Dhyana 400 DC digital camera (Tucsen Photonics, China) with Leica S8AP0 stereomicroscope (Leica Micro systems, Germany). Photographs were stacked using Helicon Focus software (v. 8.2.2. Pro, Helicon Soft, Ukraine) and stacked digital images were taken using Adobe Photoshop 2025 (v. 25.12.4, Adobe, USA).

## Taxon treatments

### Pseudoformicaleo

van der Weele, 1909

052781D3-9711-5B4C-98A8-A7F793938FCD

Pseudoformicaleo van der Weele, 1909 - van der Weele 1909: 25. Type species: *Myrmeleon gracilis* Klug in Ehrenberg, 1834.
Tahulus Navás, 1912 - Navás 1912: 112.
Nadal Navás, 1912 - Navás 1912: 454.
Pseudoplectron Navás, 1914 - Navás 1914a: 467.

#### Diagnosis

Antenna as long as head plus thorax length. Eye large, wider than frons. Pronotum longer than wide. Forewing vein RP arising beyond CuA fork. Hindwing with one presectoral crossvein; basal half of hindwing vein CuA runs parallel with hind margin. Leg thick; tarsomere 1 very long, at least as long as tarsomere 5; tibial spurs straight, as long as tarsomere 1; tarsal claw medially with a tooth-like projection (Balakrishnan et al. 2024).

#### Distribution

Palaearctic (Algeria, China, Egypt, Iran, Israel, Japan, Lebanon, Libya, Morocco, Palestine, Russia, Saudi Arabia, Syria, Tunisia, Turkey, United Arab Emirates, Yemen), Oriental (India, Indonesia, Malaysia, Pakistan, Sri Lanka, Thailand, Vietnam), Afrotropical (Benin, Kenya, Madagascar, Oman), Australian (Australia, Palau, Papua New Guinea) (Sekimoto 2014, Balakrishnan et al. 2024, Oswald 2026).

### Pseudoformicaleo nubecula

(Gerstaecker, 1885)

6F605FA8-9163-5C21-BA90-11AE6E0B634E

Creagris nubecula Gerstaecker, 1885 - Gerstaecker 1884: 101. Type locality: Rockhampton, Australia.
Pseudoformicaleo jacobsoni van der Weele, 1909 - van der Weele 1909: 25.
Pseudoformicaleo jacobsoni wetterensis van der Weele, 1909 - van der Weele 1909: 27.
Creagris matsuokae Okamoto, 1910 - Okamoto 1910: 282.
Protoplectron costatus Banks, 1910 - Banks 1910: 41.
Tahulus caligatus Navás, 1912 - Navás 1912: 113.
Creagris horikawae Nakahara, 1913 - Nakahara 1913: 527.
Pseudoplectron costatus (Banks, 1910): Navás 1914b: 467.
Tahulus asthenicus Navás, 1914 - Navás 1914c: 140.
Tahulus ignobilis Navás, 1914 - Navás 1914b: 115.
Gama matsuokae (Okamoto, 1910): Banks 1937: 287.
Exiliunguleon nanus Yang, 1999 - Yang 1999: 154.
Creoleon matsuokae (Okamoto, 1910): Lin et al. 2019: 136.
Pseudoformicaleo asthenicus (Navás, 1914): Lin et al. 2019: 136.
Pseudoformicaleo caligatus (Navás, 1912): Lin et al. 2019: 136.
Pseudoformicaleo nubecula (Gerstaecker, 1885): Esben-Petersen 1915: 67; Esben-Petersen 1920: 191; New 1985: 43; Stange et al. 2003: 102; Stange 2004: 122; Krivokhatsky et al. 2012: 573; Yoshitomi et al. 2013: 3; Sekimoto 2014: 71; Matsumoto et al. 2016: 107; Wang et al. 2018: 139; Balakrishnan et al. 2024: 3.

#### Materials

- **Type status:** Other material. **Occurrence:** recordedBy: Minkyu Jeong; individualCount: 1; sex: 1 male; lifeStage: adult; occurrenceID: BFF2D947-EA79-5871-A861-B46CB74D347D; **Taxon:** scientificName: *Pseudoformicaleo nubecula*; **Location:** country: South Korea; stateProvince: Jeollanam-do; locality: Shinan-gun, Bigeum-myeon, Naewol-ri; **Identification:** identifiedBy: Jiseung Kim; **Event:** eventDate: 2024-07-27; **Record Level:** institutionCode: JBNU
- **Type status:** Other material. **Occurrence:** recordedBy: Jiseung Kim and Jaegwan Yang; individualCount: 3; sex: 2 males, 1 female; lifeStage: adult; occurrenceID: 7D09068B-44FE-51C4-AE46-7F2875531F58; **Taxon:** scientificName: *Pseudoformicaleo nubecula*; **Location:** country: South Korea; stateProvince: Jeollanam-do; locality: Shinan-gun, Bigeum-myeon, Naewol-ri; verbatimElevation: 28 m; decimalLatitude: 34.752081; decimalLongitude: 125.896569; **Identification:** identifiedBy: Jiseung Kim; **Event:** eventDate: 2025-08-13; **Record Level:** institutionCode: JBNU

#### Description

**Male, adult**.

**Figure 1.**
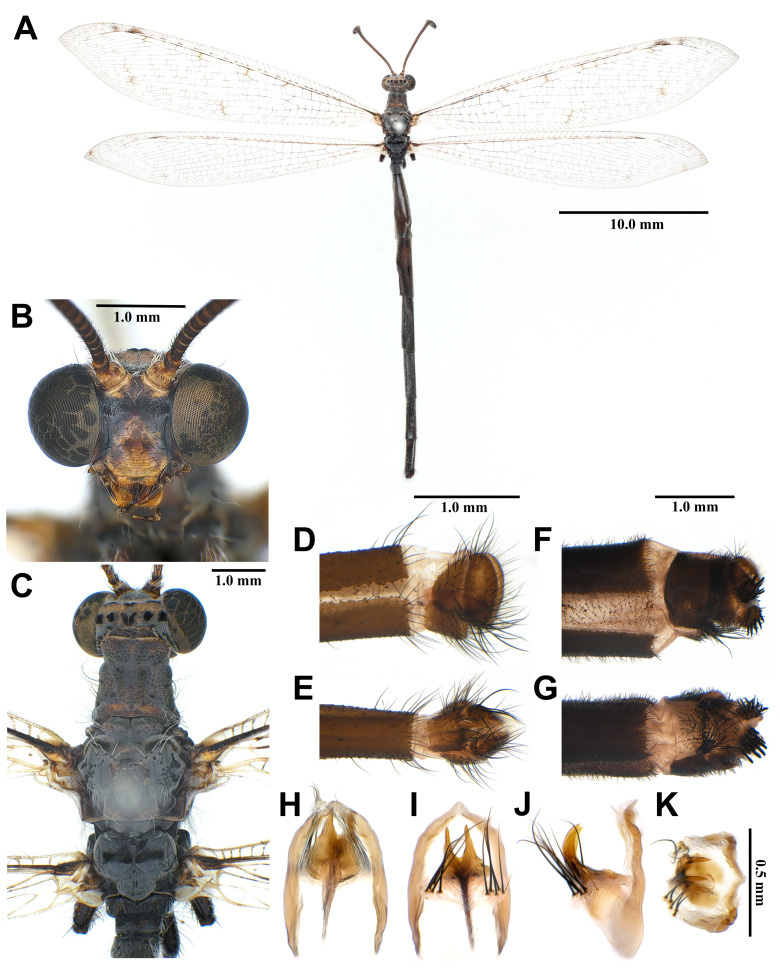
*Pseudoformicaleo nubecula* (Gerstaecker), adult. **A** Dorsal habitus, male; **B** head, frontal view; **C** head and thorax, dorsal view; **D, E** male terminalia: lateral view (**D**) and ventral view (**E**); **F, G** female terminalia: lateral view (**F**) and ventral view (**G**); **H, K** male genitalia: dorsal view (**H**) and ventral view (**K**); **J** lateral view; **K** caudal view.

**Head** (Fig. 1B, C). Vertex slightly narrow, moderately raised, dark gray, with two rows of dark spots. Frons yellow, with broad dark brown marking surrounding eye and base of antenna, with sparse dark setae; clypeus yellow, with long pale yellow hairs. Antenna dark brown, slightly short, with well defined club, densely covered with short black hairs; flagellum comprising approximately 38 flagellomeres, each flagellomere with distal yellow annulation. Labrum yellow, with short black setae; maxillary palpus dark brown; labial palpus yellowish brown.

**Thorax** (Fig. 1C). Pronotum narrow, length longer than width, dark gray, with long black and white hairs. Mesonotum dark gray, with long white hairs. Metanotum dark gray, with dark transverse stripe at middle, with short white hairs.

**Legs**. Coxae mostly gray, moderately covered with long white hairs. Femora mostly dark brown, partly yellowish brown, moderately covered with black and white hairs. Tibiae mostly dark brown, partly yellowish brown, moderately covered with long black and white hairs. Tibial spurs yellowish brown, long, slightly curved, approximately as long as tarsomere 1. Tarsi dark brown; tarsomere 1 much longer than combined lengths of tarsomeres 2–4; tarsomere 5 approximately as long as tarsomere 1. Claws reddish brown, approximately as long as combined lengths of tarsomeres 2–4, toothed.

**Wings** (Fig. 1A). With dark brown markings. Forewings veins and crossveins dark brown and pale yellow; presectoral area with six or seven crossveins; RP arising beyond CuA fork; CuP supporting one cell before fusing with 1A; 2A and 3A separate; pterostigma white, with proximal dark brown spot; anterior Banksian lines absent; posterior Banksian lines absent. Hindwing shorter and narrower than forewing; presectoral area with one crossvein; RP arising before MP fork; pterostigma white; anterior Banksian lines absent; posterior Banksian lines absent.

**Figure 2.**
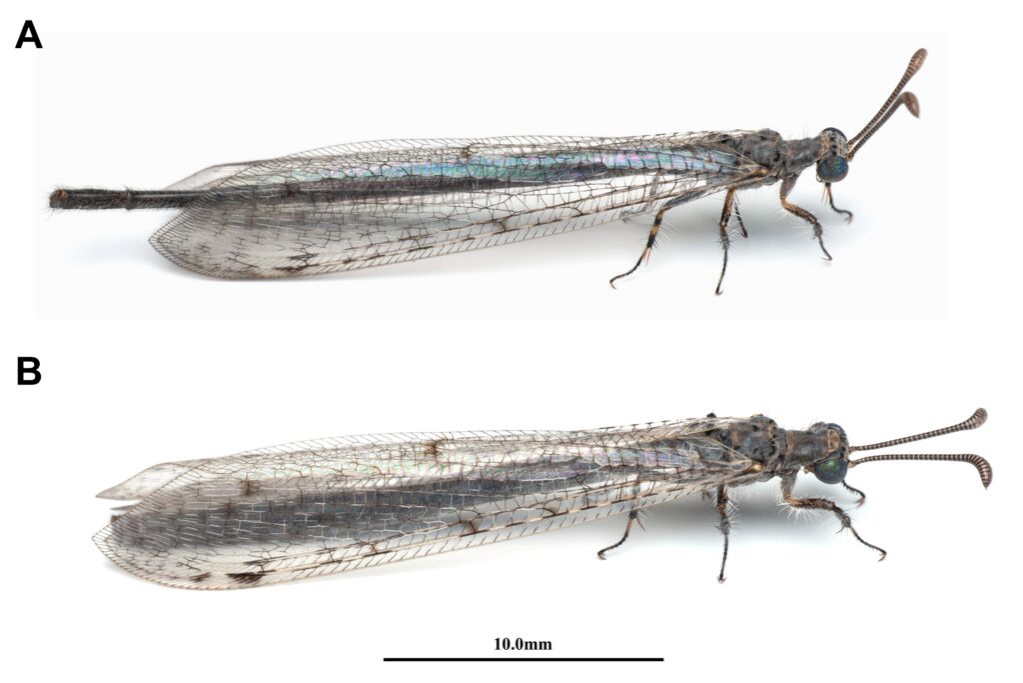
*Pseudoformicaleo nubecula* (Gerstaecker), living adult. **A** Male; **B** female.

**Abdomen** (Fig. 1A, Fig. 2A). Longer than hindwing, dark gray, densely covered with short black and white setae.

**Genitalia** (Fig. 1D, E, H–K). Ectoproct semicircular, covered with long black setae. Sternite IX narrow, triangular, covered with long black setae. Gonarcus brown, arched. Mediuncus absent. Parameres well sclerotized, dark brown, with long black setae, moderately hooked in lateral view.

**Size**. BL: 29.1–30.5 mm; FWL: 22.1–24.1 mm; HWL: 20.1–21.4 mm.

**Female, adult**.

Except length of abdomen and terminalia, generally similar to male. Female abdomen shorter than hindwing (Fig. 2B). Terminalia (Fig. 1F, G): tergite VIII wider than tergite IX; tergite IX narrow, rectangular in lateral view; ectoproct semicircular in lateral view; lateral gonapophyses semicircular in lateral view, smaller than ectoproct; posterior gonapophyses short, with long black setae; anterior gonapophyses absent; pregenital plate absent.

**Size**. BL: 26.3 mm; FWL: 23.8 mm; HWL: 21.7 mm.

#### Diagnosis

*Pseudoformicaleo nubecula* is the smallest myrmeleontid species in Korea, with the male abdomen being shorter than the hindwing. In the forewing, 1A and 2A lack crossveins, RP has 11–12 radial veins, and pterostigma has proximal dark brown spot. In the hindwing, RP has 10 radial veins.

#### Distribution

Korea (new record), Australia, China, Indonesia, Japan, Malaysia, Palau, Papua New Guinea, Sri Lanka, Vietnam, India, Thailand (Sekimoto 2014, Balakrishnan et al. 2024).

#### Ecology

**Figure 3.**
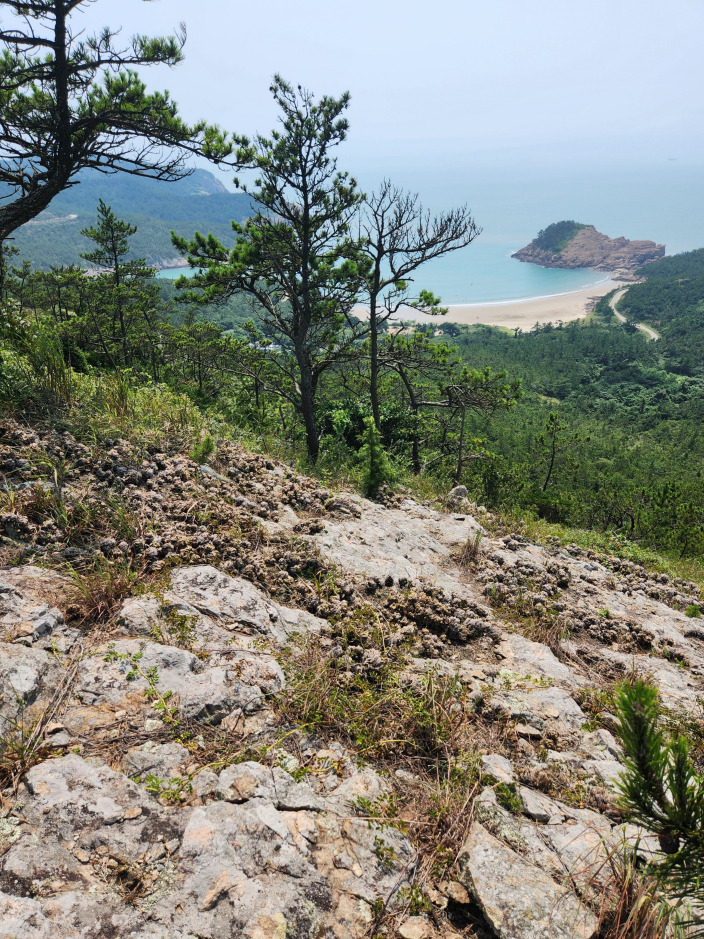
Habitat. Rocky hill and coastal forest behind the natural coastal dune (Naewol-ri).

*Pseudoformicaleo nubecula* was observed in a rocky hill resting on plant stems at night (Fig. 3). Larvae are known to be ambush hunters but were not examined during this study; for details on their ecology, refer to Matsumoto et al. (2016).

#### Notes

*Pseudoformicaleo nubecula* has a very broad distribution extending from Asia to Australia and is known to show various morphological variations, so additional molecular research is required (Balakrishnan et al. 2024).

Suggested vernacular Korean name is “Kko-ri-myeong-ju-jam-ja-ri”, referring to the long abdomen of the male.

### Paraglenurus

van der Weele, 1909

E4266A11-66E4-5767-8A67-3FA0772A9858

Paraglenurus van der Weele, 1909 - van der Weele 1909: 29. Type species: *Myrmeleon scopifer* Gerstaecker, 1887.
Glenuroides Okamoto, 1910 - Okamoto 1910: 294.
Eoleon Navás, 1921 - Navás 1921: 65.

#### Diagnosis

Antenna longer than head plus thoracic length. Eye large, nearly as wide as frons. Leg slender; hind femur plus tibia nearly as long as combined length of head plus thorax; pretarsal claw opposable. Male paramere curved slender plate-like; mediuncus prominent. Female tergite VII with some thick setae on posterior margin; posterior gonapophyses elongate, digitiform; lateral gonapophyses covered with thick digging setae (Zheng and Liu 2025).

#### Distribution

Palaearctic (China, Japan, Korea, Russia (Far East)), Oriental (Indonesia, Vietnam), Afrotropical (Madagascar, Seychelles) (Sekimoto 2014, Ábrahám 2023, Zheng and Liu 2025).

### Paraglenurus okinawensis

(Okamoto, 1910)

CB7B906D-2753-5370-AD22-501CE7E7B684

Glenuroides okinawensis Okamoto, 1910 - Okamoto 1910: 296. Type locality: Okinawa, Japan.
Paraglenurus okinawensis (Okamoto, 1910): Miller et al. 1999: 58. Sekimoto 2014: 67; Ikeda and Okui 2017: 20; Hoshino 2019: 16; Matsumoto et al. 2021: 7.

#### Materials

- **Type status:** Other material. **Occurrence:** recordedBy: Jiseung Kim and Jaegwan Yang; individualCount: 4; sex: 2 males, 2 females; lifeStage: adult; occurrenceID: 55BCF082-33E2-5F88-A810-1EC6D6EEA64C; **Taxon:** scientificName: *Paraglenurus okinawensis*; **Location:** country: South Korea; stateProvince: Jeollanam-do; locality: Shinan-gun, Bigeum-myeon, Naewol-ri; verbatimElevation: 28 m; decimalLatitude: 34.752081; decimalLongitude: 125.896569; **Identification:** identifiedBy: Jiseung Kim; **Event:** eventDate: 2025-08-13; **Record Level:** institutionCode: JBNU

#### Description

**Male, adult**.

**Figure 4.**
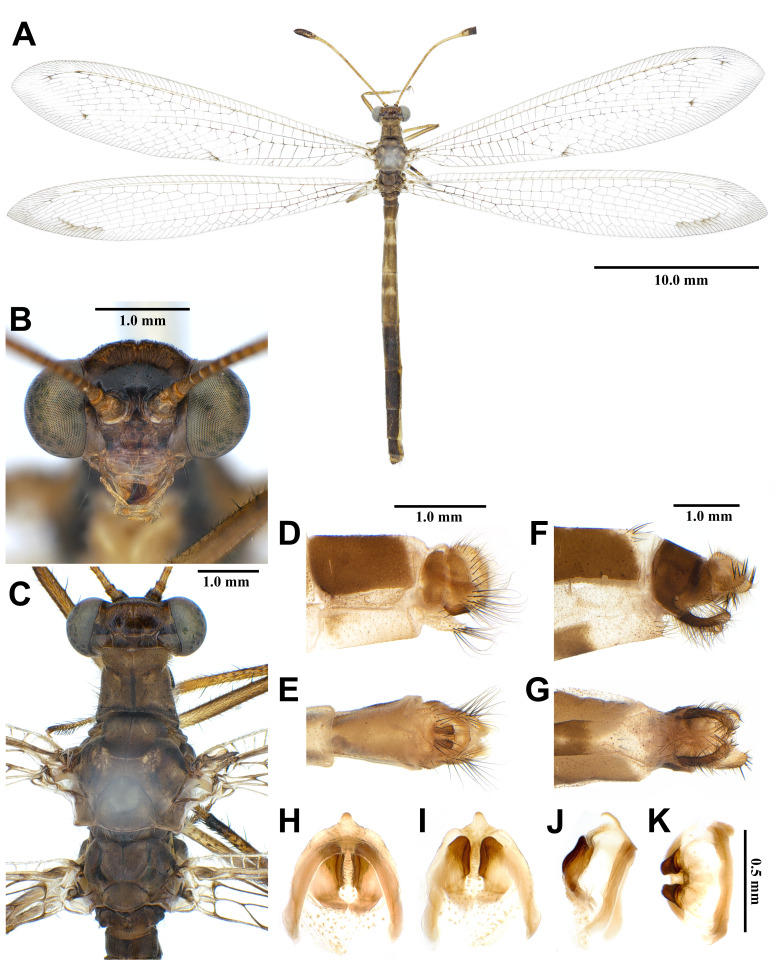
*Paraglenurus okinawensis* Okamoto, adult. **A** Dorsal habitus, male; **B** head, frontal view; **C** head and thorax, dorsal view; **D, E** male terminalia: lateral view (**D**) and ventral view (E); **F, G** female terminalia: lateral view (**F**) and ventral view (**G**); **H, K** male genitalia: dorsal view (**H**) and ventral view (**I**); **J** lateral view; **K** caudal view.

**Head** (Fig. 4B, C). Vertex wide, moderately raised, brown. Frons brown, with broad dark brown band extending from below vertex to below base of antenna; clypeus pale yellow, with hyaline brown hairs. Antenna pale brown, long, with slightly defined club, densely covered with short black hairs; flagellum comprising approximately 45 flagellomeres. Mouthparts pale brown; labrum pale yellow, with hyaline brown hairs; maxillary palpus pale yellow; labial palpus pale yellow, spindle-shaped.

**Thorax** (Fig. 4C) Pronotum slender, length longer than width, brown, with long black and hyaline brown hairs. Mesonotum and metanotum brown, with sparse black and hyaline brown hairs.

**Legs**. Coxae yellow, moderately covered with long white hairs. Femora mostly yellow, partly brown, moderately covered with black hairs. Tibiae yellow, partly dark brown, moderately covered with black hairs. Tibial spurs yellowish brown, slightly long, slightly curved, approximately as long as tarsomere 1. Tarsi yellow; tarsomere approximately as long as combined lengths of tarsomeres 2–3; tarsomere 5 longer than tarsomere 1. Claws yellowish brown, approximately as long as tibial spurs.

**Wings** (Fig. 4A). With white and dark brown markings. Forewings veins and crossveins dark brown and white; rhegma area with small brown spot; presectoral area with 9–11 crossveins; RP arising beyond CuA fork; CuP supporting one cell before fusing with 1A; 2A fused with 3A; pterostigma white; anterior Banksian lines absent; posterior Banksian lines absent. Hindwing as long as forewing; veins and crossveins dark brown; rhegma area with larger brown marking; presectoral area with one crossvein; RP arising before MP fork; pterostigma white; anterior Banksian lines absent; posterior Banksian lines absent.

**Abdomen** (Fig. 4A). Shorter than hindwing, brown, posterior margin of tergites II–VII bordered with yellow, tergites III–V with median yellow marking, densely covered with brown hairs.

**Genitalia** (Fig. 4D, E, H–K). Ectoproct semicircular, covered with long black setae. Sternite IX narrow, covered with sparse brown setae. Gonarcus brown, arched. Mediuncus brown, lightly sclerotized, lightly hooked in lateral view. Parameres well-sclerotized, dark brown, triangular in caudal view.

**Size**. BL: 20.3–20.9 mm; FWL: 21.4–22.2 mm; HWL: 21.3–22.1 mm.

**Female, adult**.

Except terminalia, generally similar to male. Terminalia (Fig. 4F, G): tergite VIII wider than tergite IX; tergite IX narrow, triangular in lateral view; ectoproct triangular in lateral view; lateral gonapophyses with long black setae; posterior gonapophyses long, curved, with densely covered with long black setae; anterior gonapophyses absent; pregenital distinct, plate triangular, presented on membrane below tergite VIII.

**Size**. BL: 20.6–22.1 mm; FWL: 24.1–26.3 mm; HWL: 24.0–26.3 mm.

#### Diagnosis

*Paraglenurus okinawensis* is a small species compared to its congeners, with the male forewing length not exceeding 24 mm. The head morphology is similar in both sexes. The fore femur is slender, approximately 10 times as long as thick. The preapical dark marking on the hindwing is linear and parallel to the posterior margin.

#### Distribution

Korea (new record), Japan (Matsumoto et al. 2021).

#### Ecology

*Paraglenurus okinawensis* was collected in a coastal forest behind the natural dune during our survey (Fig. 3). Larvae are known to be ambush hunters but were not examined during this study; for details on their ecology, refer to Matsumoto et al. (2021).

#### Notes

Unlike other species of the same genus that show a relatively broad distribution across Korea, to date, *Paraglenurus okinawensis* has only been collected near the coast of Bigeumdo.

Suggested vernacular Korean name is “Kko-ma-byeol-bak-i-myeong-ju-jam-ja-ri”, referring to it as the smallest species of the same genus recorded in the Korean Peninsula.

## Identification Keys

### Key to species of Nemoleontinae in Korea

**Table:** 

1	Antenna approximately as long as length of head plus thorax. Eye small, narrower than frons. Leg thick, hind femur plus tibia shorter than length of head plus thorax; claw not opposable. Male genitalia with a forked paramere (tribe Nemoleontini)	2
–	Antenna longer than length of head plus thorax. Eye big, as wide as frons. Leg slender, hind femur plus tibia approximately as long as length of head plus thorax; claw opposable. Male genitalia with a pair of plate-like paramere (tribe Megistopini, genus *Paraglenurus*)	5
2	Hindwing presectoral area with 2 crossveins (genus *Deutoleon*)	*Deutoleon lineatus* (Fabricius)
–	Hindwing presectoral area with only 1 crossvein	3
3	Smaller in size, forewing length ~22–24 mm. Abdomen longer than hindwing in male (genus *Pseudoformicaleo*)	*Pseudoformicaleo nubecula* ()
–	Larger in size, forewing length ~29–44 mm. Abdomen shorter than hindwing in male (genus *Distoleon*)	4
4	3rd labial palpomere dark brown. Hindwing rhegma area with distinct large dark brown marking	*Distoleon nigricans* (Okamoto)
–	3rd labial palpomere yellowish brown. Hindwing rhegma area without distinct large dark brown marking	*Distoleon littoralis* Miller & Stange
5	Preapical dark marking at rhegma in hindwing linear, parallel to hind margin	*Paraglenurus okinawensis* (Okamoto)
–	Preapical dark marking at rhegma in hindwing distally expanded, mostly not linear, and not parallel to hind margin	6
6	Abdominal tergites II–V largely yellowish white in male. Forewing without white marking along posterior margin	*Paraglenurus albiventris* Matsumoto, Kikuta & Hayashi
–	Abdominal tergites III–V dark brown, each with pair of median pale spots. Forewing with white marking along posterior margin	7
7	Antenna apical ~1/4, each flagellum with apical 1/3–1/2 pale yellow. Hindwing with a distinct preapical dark brown marking and a distinct and rounded adjacent white marking	*Paraglenurus melanostictus* Matsumoto, Kikuta & Hayashi
–	Antenna apical ~1/4, each flagellum with apex only slightly pale yellow. Hindwing with a distinct preapical dark brown marking and an indistinct and oval-shaped adjacent white marking	*Paraglenurus japonicus* (McLachlan)

## Supplementary Material

XML Treatment for Pseudoformicaleo

XML Treatment for Pseudoformicaleo nubecula

XML Treatment for Paraglenurus

XML Treatment for Paraglenurus okinawensis
